# Supplementary figures and images for: Less is more? Ultra-low carbohydrate diet and working dogs’ performance
Source: PLoS One. 2021 Dec 23;16(12):e0261506. doi: 10.1371/journal.pone.0261506 (PMC8699952; doi:10.1371/journal.pone.0261506)

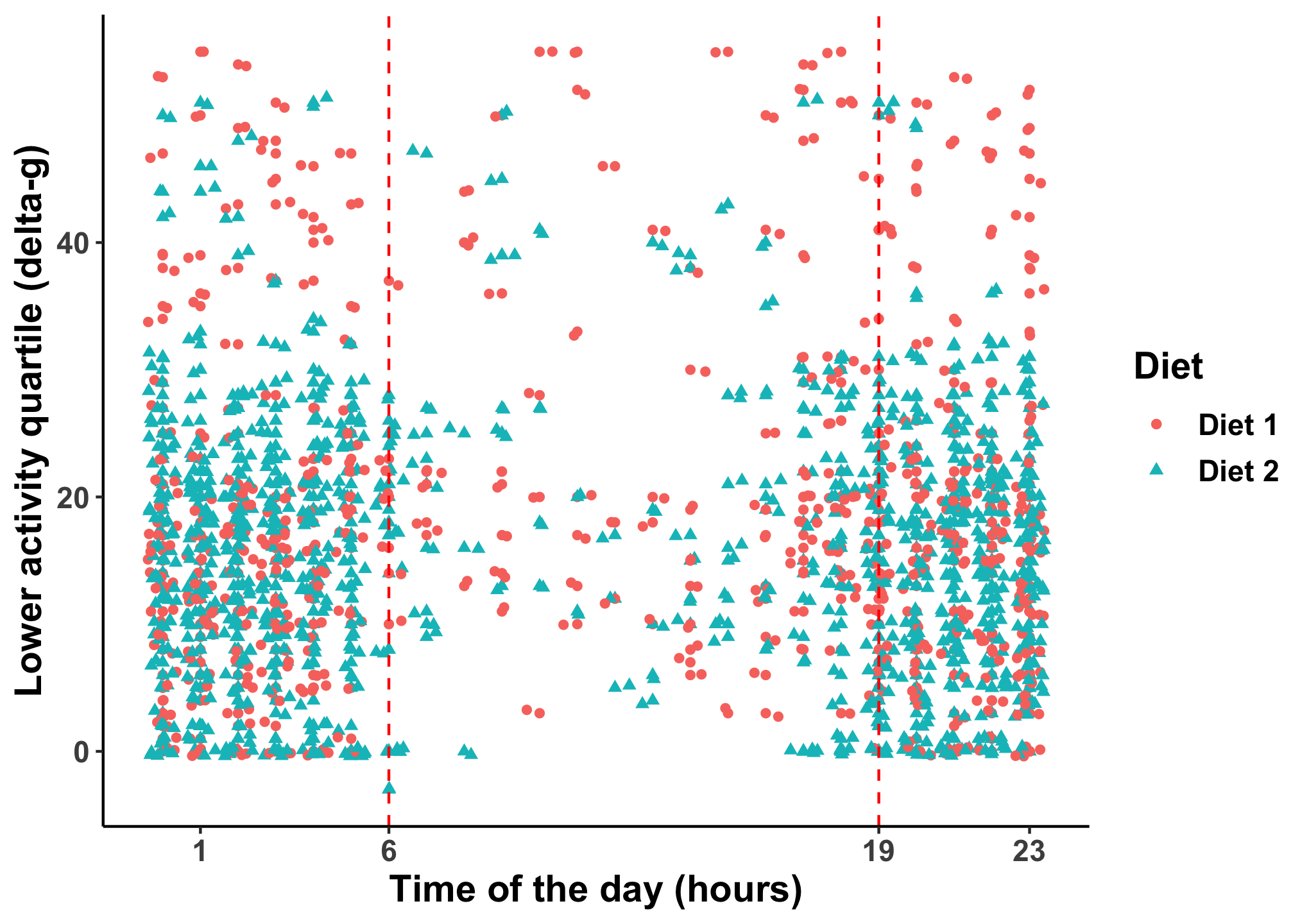

Supplement: S1 Fig — The period between the two broken red vertical lines denotes the time the dogs were awake and working. Diet 1, high %ME CHO low %ME fat diet; Diet 2, ultra-low %ME CHO high %ME fat diet. (TIF) [file pone.0261506.s001.tif]

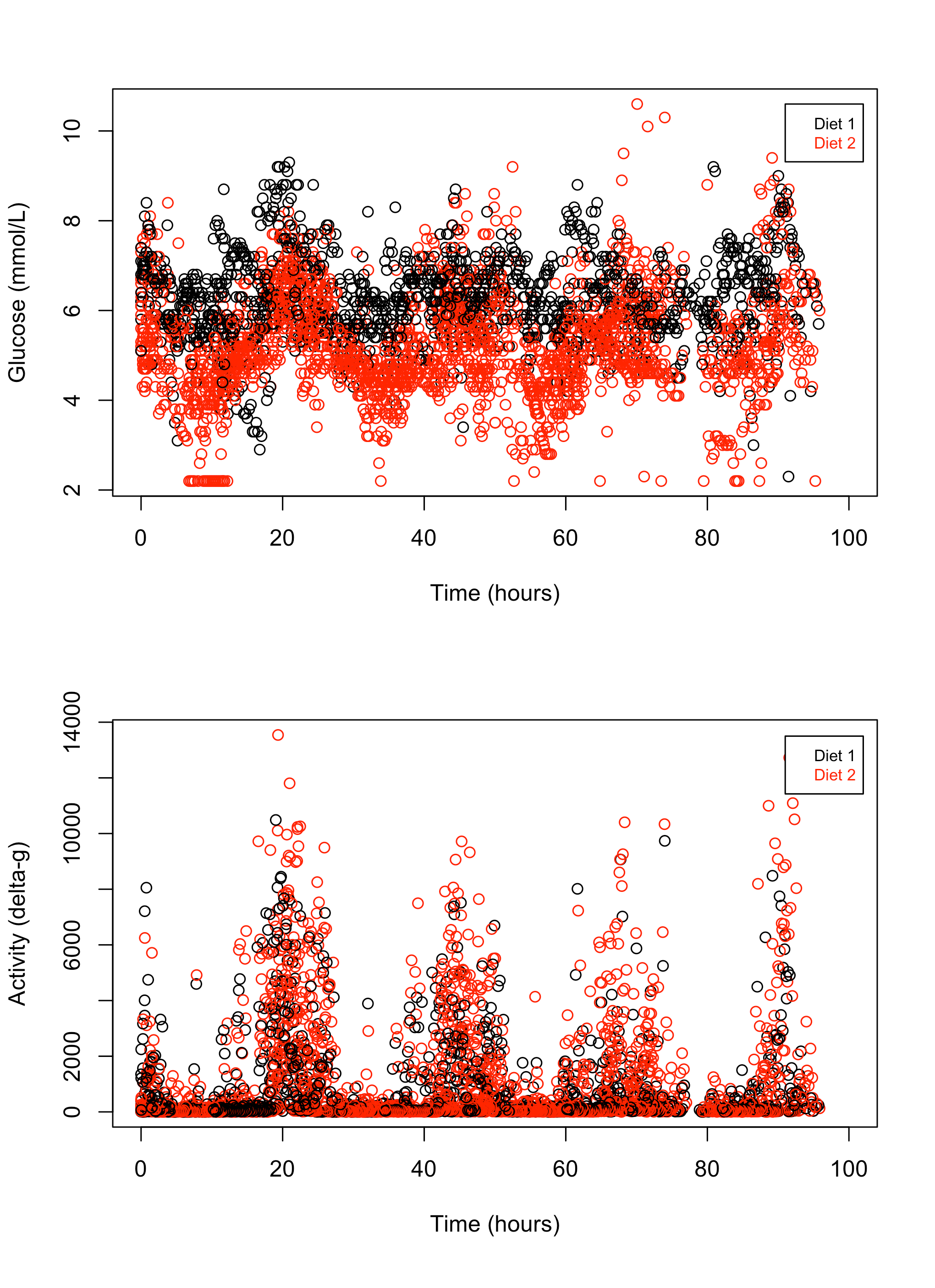

Supplement: S2 Fig — Diet 1, high %ME CHO low %ME fat diet; Diet 2, ultra-low %ME CHO high %ME fat diet. (TIF) [file pone.0261506.s002.tif]

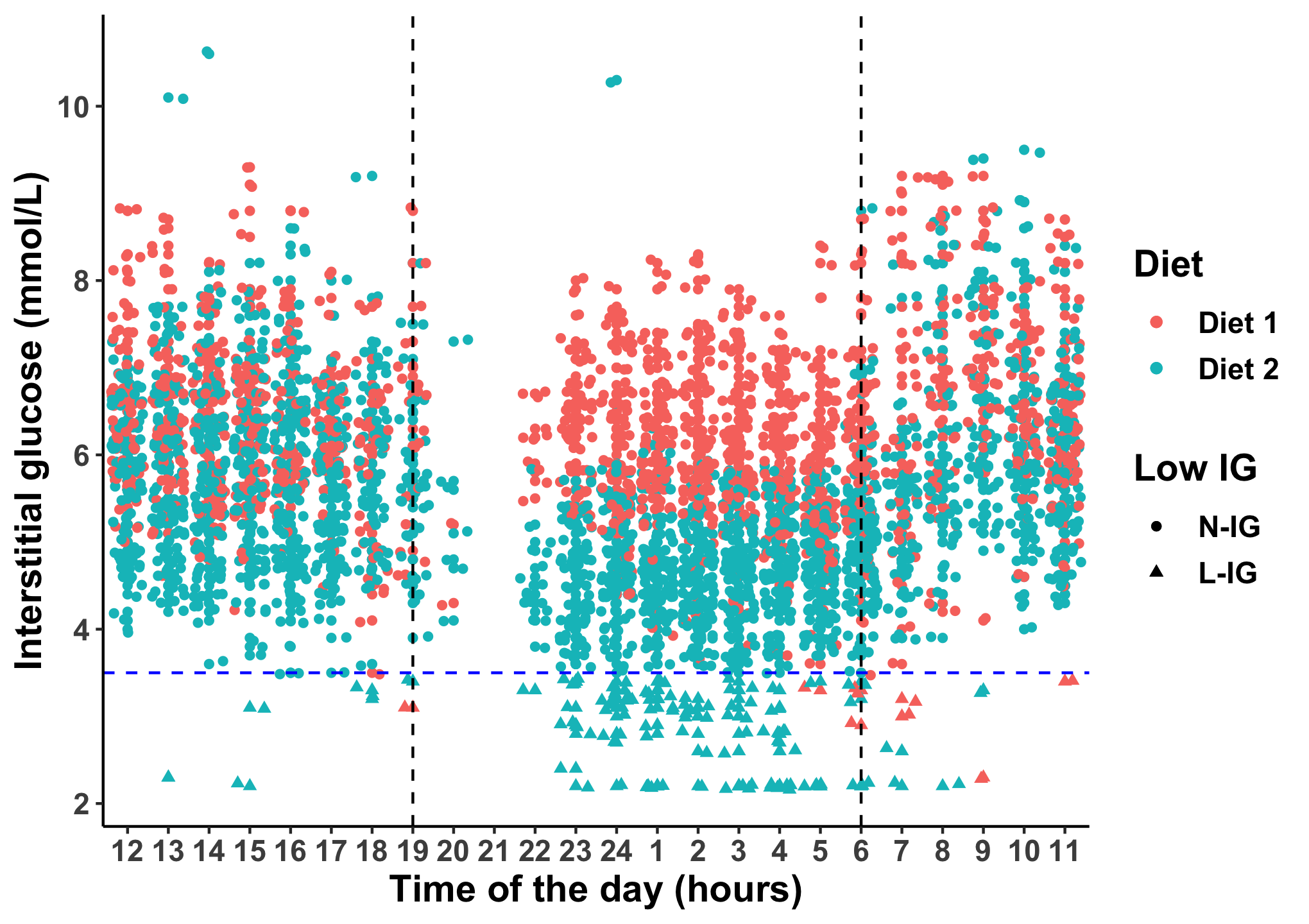

Supplement: S3 Fig — Diet 1, high %ME CHO low %ME fat diet; Diet 2, ultra-low %ME CHO high %ME fat diet; IG, interstitial glucose; L-IG, low interstitial glucose (<3.5 mmol/L); N-IG, normal interstitial glucose (3.5 mmol– 5.6 mmol/L). The horizontal dashed blue line separates the L-IG (below) from the N-IG (above). The vertical broken black lines denote the period between 19:00–06:00. (TIF) [file pone.0261506.s003.tif]

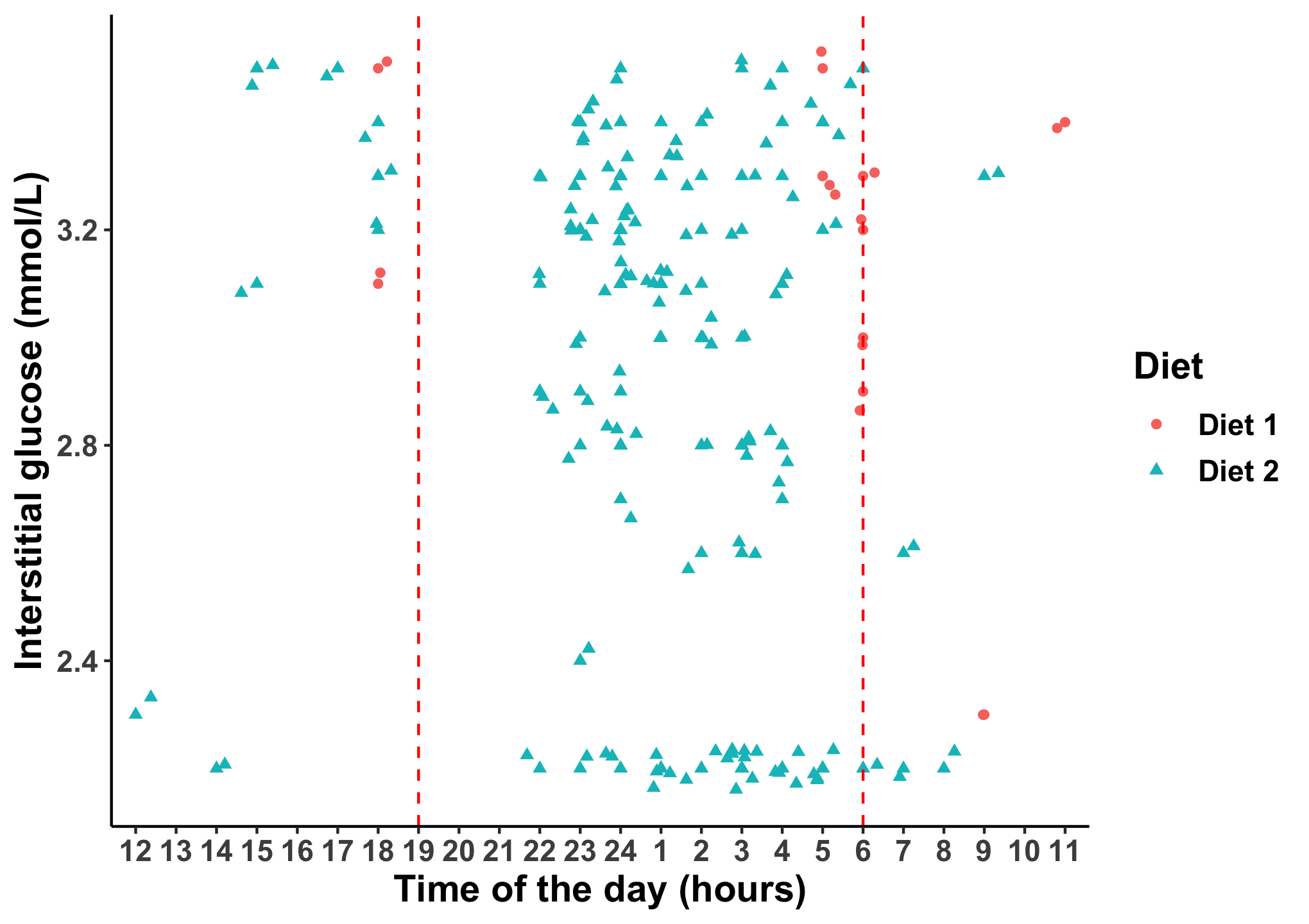

Supplement: S4 Fig — Diet 1, high %ME CHO low %ME fat diet; Diet 2, ultra-low %ME CHO high %ME fat diet. The broken red lines denote the period between 19:00–06:00. (TIF) [file pone.0261506.s004.tif]
