# Supplementary material for: Less is more? Ultra-low carbohydrate diet and working dogs’ performance
Source: PLoS One. 2021 Dec 23;16(12):e0261506. doi: 10.1371/journal.pone.0261506 (PMC8699952; doi:10.1371/journal.pone.0261506)
Supplement: S1 Table — (DOCX) [file pone.0261506.s005.docx]

| **S1 Table.** Nutritional analysis of trial diets | |  |
| --- | --- | --- |
| Percentage by weight | Diet 1 | Diet 2 |
| Crude protein | 22 | 51.6 |
| Crude fat | 10 | 36.4 |
| NFE (by difference) | 51 | 1.1 |
| Crude fibre | 2 | 2.1 |
| Ash | 8 | 5.2 |
| Moisture | 7 | 3.6 |
|  |  |  |
| % ME |  |  |
| Protein | 23 | 37 |
| Fat | 25 | 63 |
| Carbohydrate | 52 | 1 |
|  |  |  |
| Energy density (kcal/g) | 3.4 | 4.9 |
