# Supplementary material for: Less is more? Ultra-low carbohydrate diet and working dogs’ performance
Source: PLoS One. 2021 Dec 23;16(12):e0261506. doi: 10.1371/journal.pone.0261506 (PMC8699952; doi:10.1371/journal.pone.0261506)
Supplement: S2 Table — (DOCX) [file pone.0261506.s006.docx]

| **S2 Table.** Ingredients of trial diets. | |
| --- | --- |
| **Diet** | **Ingredients** |
| Diet 1^†^ | Cereals &/or cereal by-products, meat & meat by-products (poultry, beef &/or lamb), poultry palatant, beet pulp, iodised salt, minerals (iron, zinc, copper, potassium and selenium), sunflower oil, vitamins (A, D3, E, B1, B2, B3, B5, B6, B9, B12 and choline), amino acid, antioxidants |
| Diet 2* | Chicken, Eggs, Flaxseed Flakes, Hoki Oil, Brown Kelp, New Zealand Green Mussel, Pumpkin, Broccoli, Cauliflower, Cabbage, Potassium Sulphate, Dried Kelp, Apples, Pears, Salt, Vitamin E Supplement, Zinc Proteinate, Iron Proteinate, Sunflower Oil, Magnesium Oxide, Selenium Yeast, Copper Proteinate, Manganese Proteinate, Beta-Carotene, Thiamine Mononitrate, Vitamin D3 Supplement. |
| ^†^Diet 1 Pedigree Adult real chicken®, MARS Petcare New Zealand, Auckland, New Zealand  *Diet 2 Chicken Feast Freeze Dried®, K9 Natural, Christchurch, New Zealand | |
