# Supplementary material for: Less is more? Ultra-low carbohydrate diet and working dogs’ performance
Source: PLoS One. 2021 Dec 23;16(12):e0261506. doi: 10.1371/journal.pone.0261506 (PMC8699952; doi:10.1371/journal.pone.0261506)
Supplement: Attachment — Submitted filename: Response to Reviewers.docx [file pone.0261506.s007.docx]

The authors thank the Editor and the Reviewer for the time they invested in reviewing the manuscript and providing their valuable comments and suggestions for improving the manuscript and making it more impactful. Below we provide a point-by-point responses to each of the comments made by the Editor and Reviewer.

Journal Requirements:

When submitting your revision, we need you to address these additional requirements.

1. Please ensure that your manuscript meets PLOS ONE's style requirements, including those for file naming. The PLOS ONE style templates can be found at

<https://journals.plos.org/plosone/s/file?id=wjVg/PLOSOne_formatting_sample_main_body.pdf> and

[https://journals.plos.org/plosone/s/file?id=ba62/PLOSOne_formatting_sample_title_authors_affiliations.pdf](about:blank)

AUTHORS’ response: Files were named according to journal’s requirements.

2. In your Methods section, please provide additional details regarding participant consent from the owners of the animals. In the ethics statement in the Methods and online submission information, please ensure that you have specified (a) whether consent was informed and (b) what type you obtained (for instance, written or verbal). If the need for consent was waived by the ethics committee, please include this information.

AUTHORS’ response: We added the following sentence in the methods section (L86-87): “Dogs were recruited after receipt of owner’s verbal consent to participate in the study.”

3.  Thank you for stating the following in the Competing Interests section:

"The authors have declared that no competing interests exist."

We note that one or more of the authors are employed by a commercial company: "VetEnt Te Kuiti, Pet Doctors and North Coast Veterinary Specialist and Referral Centre"

a) Please provide an amended Funding Statement declaring this commercial affiliation, as well as a statement regarding the Role of Funders in your study. If the funding organization did not play a role in the study design, data collection and analysis, decision to publish, or preparation of the manuscript and only provided financial support in the form of authors' salaries and/or research materials, please review your statements relating to the author contributions, and ensure you have specifically and accurately indicated the role(s) that these authors had in your study. You can update author roles in the Author Contributions section of the online submission form.

Please also include the following statement within your amended Funding Statement.

“The funder provided support in the form of salaries for authors [insert relevant initials], but did not have any additional role in the study design, data collection and analysis, decision to publish, or preparation of the manuscript. The specific roles of these authors are articulated in the ‘author contributions’ section.”

If your commercial affiliation did play a role in your study, please state and explain this role within your updated Funding Statement.

AUTHORS’ response:  Dr. Burchell was employed at Massey University during the time of the study. He left after the study was completed and has been working in a private referral center. His current employment had no impact on the study whatsoever. Like many private practitioners involved in research his involvement in the paper subsequent to leaving the university sector has been in his own time. Drs. Giles and Cuttance were not employed at Massey University at the time of the study. Their role in the study is articulated in the Authors’ Contributions sections and they did not have any conflict of interests with regards to the aforementioned study.

We added the requested sentence in L441-445 and in the online submission system.

b) Please also provide an updated Competing Interests Statement declaring this commercial affiliation along with any other relevant declarations relating to employment, consultancy, patents, products in development, or marketed products, etc.

Within your Competing Interests Statement, please confirm that this commercial affiliation does not alter your adherence to all PLOS ONE policies on sharing data and materials by including the following statement: "This does not alter our adherence to  PLOS ONE policies on sharing data and materials.” (as detailed online in our guide for authors [http://journals.plos.org/plosone/s/competing-interests](about:blank)) . If this adherence statement is not accurate and  there are restrictions on sharing of data and/or materials, please state these. Please note that we cannot proceed with consideration of your article until this information has been declared.

Please include both an updated Funding Statement and Competing Interests Statement in your cover letter. We will change the online submission form on your behalf.

AUTHORS’ response: We did not find anywhere in PLOS ONE Editorial system during resubmission a place where we could amend the Competing Interests Statement. In the ‘manuscript data tab’ under ‘Funding information’ there was no area to amend what the editor requested. The roles of each contributor accurately reflect their involvement at different aspects that were listed.

Reviewers' comments:

Reviewer #1: First, I would like to thank the author for their interesting manuscript. The work described by the authors sheds more light on the effects of macronutrient composition in dogs performing strenuous exercise. I have a few general comments for the authors, followed by some specific remarks.

- The manuscript is quite long, and some paragraphs have a lot of repetition in them. I think the readability of the manuscript may be improved by optimizing conciseness throughout the work.

AUTHORS’ response: We followed the reviewer suggestions and trimmed the parts in which there was unnecessary repetition.

- Please double check the consistency of your abbreviation use throughout the manuscript. After introducing an abbreviation, you sometimes switch back to the fully written alternative.
AUTHORS’ response: We made the suggested change.

- The discussion section may benefit from some editing. I think it is important to focus foremost on the primary goals of this study, i.e. to determine whether IG concentrations and physical activity differ between diets, before focusing on your secondary findings, such as the moments of low IG that you found. You did this well in the abstract, so I would consider using the same order in your discussion.
AUTHORS’ response: We restructured the discussion similar to the sequence in the abstract, as suggested by the reviewer.

- Additionally, there is a lot of focus on the cause of your findings and comparisons with previous studies, but it is also important to emphasize the consequences of your findings: what do these findings mean for working dogs? Do we all need to change their diet, or do we need to perform further specific studies? Can we extrapolate these results to all other working dogs?
AUTHORS’ response: We followed the reviewer suggestions (see below).

For the question “what do these findings mean for working dogs?”

L321-323: “As we suggested above, feeding dogs a diet of ultra-low %ME CHO and high %ME fat leads to adaptations that offer significant advantages to dogs that perform endurance work, such as New Zealand farm dogs”.

L327-329: “The adaptation to high fat diets has been postulated to be beneficial because the higher albumin, total calcium, and magnesium protects against exercise-induced hypovolemia and the adaptation to high rates of muscle fat oxidation protects against muscle injury”.

Do we all need to change their diet, or do we need to perform further specific studies? Can we extrapolate these results to all other working dogs?

L330-337: “The results of this study may not directly translate to other kinds of working dogs. For example, it is unlikely that racing dogs (e.g., Greyhounds) would benefit from ultra-low CHO high fat diet as their race-day performance relies on high levels of muscle glycogen for short anerobic bursts of high-speed running. Similarly, personal assistance dogs (e.g., seeing eye dogs) that are not expected to engage in high endurance activities might not necessary benefit from the advantages of fat adaptation like sled, military/police, search and rescue, and New Zealand farm dogs would. Maximizing the dietary benefits for each of the different fields of activity that dogs engage in our lives would require specific research tailored to the requirements of the individual field”.

- The conclusions section may also include some emphasis on why this work was important for the population you researched.
AUTHORS’ response: Identifying whether the increased activity that we found is translated to increased work performances in the field should really be the next step before making a recommendation to change the diet for all working dogs.

We added the following sentence (L438-439): “Further work is necessary to determine if ultra-low CHO high fat diet could significantly enhance work performances in New Zealand farm working dogs”.

Abstract
- I would suggest changing the order of the abstract, by first stating the goals of the study, before stating your hypothesis.

AUTHORS’ response: We made the suggested change (L24-28).

25- ‘.. their body glucose levels that in turn..’Consider: their body glucose levels which in turn
AUTHORS’ response: The sentence was changed as follow: “We hypothesized that feeding working farm dogs an ultra-low CHO diet would reduce their IG concentrations which in turn would reduce physical activity during work” (L27-28).

25- ‘a negative effect’
Could you specify this? Maybe consider: which in turn will reduce physical activity during work
AUTHORS’ response: We made the suggested change (L28).

33- I think some editing may be required, consider: ‘Following the acclimation period, we continuously monitored IG concentrations with flash glucose monitoring devices, and activity using triaxial accelerometers for 96 h.
AUTHORS’ response: We made the suggested change (L32-34).

34- Dogs fed the Diet 2
I would rephrase as: dogs fed Diet 2
AUTHORS’ response: We made the suggested change (L34).

37- ‘Interstitial glucose’
Consider replacing with IG

AUTHORS’ response: We made the suggested change (L37).


39- Please specify your proportions: 119/3810 time points?

AUTHORS’ response: To explain what the proportion relates to we added the word ‘readings’ (L39): “The overall incidence of low IG readings (< 3.5 mmol/L) was 119/3810 (3.12%)…”

42- ‘.. was associated with increased activity, and decreased IG.’
Consider: was associated with increased activity and decreased IG concentrations.
AUTHORS’ response: We changed the sentence as follow: “We conclude that feeding Diet 2 (ultra-low CHO high fat diet) to working farm dogs was associated with increased activity despite decreased IG concentrations” (L41-42).

42- ‘Interstitial glucose’ -> IG
AUTHORS’ response: We made the suggested change (L42).

43- However, you did not check for specific macronutrient, but only for the two diets that you used. I would consider changing this to: independently of the two diets.
AUTHORS’ response: We made the suggested change (L43).

Introduction

53-56 – Do you know why these diets are commonly chosen? Would you be able to give some more background for this?
AUTHORS’ response: The answer is a combination of practical convenience, and success of a single dominant brand. Dogs were historically fed meat and offal from livestock killed on farm (“home-kill”). When home-kill was fed alone, it constituted a very low to zero carbohydrate diet.

For decades, the most popular commercial food was Tux® (Nestle-Purina), originally about 45% of ME as CHO, then more recently 35% ME (Tux Energy®).

During that time, farmers have varied the amount of home-kill and Tux fed, which ranged from 100% home-kill, through 50:50, to 100% Tux (Singh et al).

Singh, I., Tucker, L. A., Gendall, P., Rutherfurd-Markwick, K. J., Cline, J., & Thomas, D. G. (2011). Age, breed, sex distribution and nutrition of a population of working farm dogs in New Zealand: results of a cross-sectional study of members of the New Zealand Sheep Dog Trial Association. New Zealand Veterinary Journal, 59(3), 133-138. 10.1080/00480169.2011.567967

65-68 – ‘Continuous absorption of glucose…’ But this does not solely depend on dietary CHO content, as the gastrointestinal passage time and interactions with other nutrient have a great effect on this.
AUTHORS’ response: The reviewer is correct. What we meant to say was that higher dietary glucose would translate to an overall increased absorption of glucose from the GI tract compared with diets low in CHO.

We changed the sentence (L65-68) to “Therefore, feeding diets with high CHO content to dogs that are undergoing strenuous and prolonged physical exercise might support the maintenance of glucose levels for a longer time through maximizing muscle and hepatic glycogen stores and glucose absorption from the gastrointestinal tract”.

72-74 I would suggest changing the order: This study hypothesized that dogs fed an ultra %ME CHO diet will have lower body glucose levels, when compared to dogs fed a high %ME CHO diet’, to emphasize on the ultra-low diet.

AUTHORS’ response: The sentence was revised as follow (72-74): “This study tested the hypothesis that dogs fed an ultra-low CHO high fat diet will have lower body glucose levels than dogs fed a high CHO low fat diet, evidenced by lower IG levels and reduced physical activity (Fig. 1)”

Methods

86 – did you use any block randomization to ensure that all farms had an equal part of dogs in either diet 1 or 2?
AUTHORS’ response: Yes. The exact allocation of number of dog/diet/farm is evident in Table 2 under Farms. We did our best to balance the dogs between diets within farms however when you’re dealing with a small uneven number of dogs/farm it is somewhat challenging…

87: I would omit ‘(see description below)’
I would suggest changing the order in sentence 86-92: ‘Three 96- hour study periods took place during times of peak seasonal work activity, during which the dogs in each farm worked together as a team.’ I would include the dietary acclimation in the ‘diet’-section.

AUTHORS’ response: We omitted ‘(see description below)’ and changed the sentence as follow: “Acclimation of the dogs to the two diets started one month prior to the commencement of each of the three 96 h study periods, which took place during times of peak seasonal work activity, during which the dogs in each farm worked together as a team.” (L89-92)

If we had completely removed the acclimation part of the sentence, then the sentence below that explains why the study spun between Oct-Nov becomes out-of-context. We hope the reviewer finds it suitable.

92- ‘body weight’
suggestion: bodyweight
AUTHORS’ response: We made the suggested change (L95).

94-96 is there any information on the validation of both methods available?
AUTHORS’ response: Yes.

For the Flash Glucose Monitoring system:

- Corradini S, Pilosio B, Dondi F, Linari G, Testa S, Brugnoli F, Gianella P, Pietra M, Fracassi F. Accuracy of a Flash Glucose Monitoring System in Diabetic Dogs. J Vet Intern Med 2016;30:983-988.
- Del Baldo F, Canton C, Testa S, Swales H, Drudi I, Golinelli S, Fracassi F. Comparison between a flash glucose monitoring system and a portable blood glucose meter for monitoring dogs with diabetes mellitus. J Vet Intern Med 2020;34:2296-2305.
- Malerba E, Cattani C, Del Baldo F, Carotenuto G, Corradini S, Golinelli S, Drudi I, Fracassi F. Accuracy of a flash glucose monitoring system in dogs with diabetic ketoacidosis. J Vet Intern Med 2020;34:83-91.
- Silva DD, Cecci GRM, Biz G, Chiaro FN, Zanutto MS. Evaluation of a flash glucose monitoring system in dogs with diabetic ketoacidosis. Domest Anim Endocrinol 2021;74:106525.

For the Heyrex accelerometer:

- Albright JD, Seddighi RM, Ng Z, Sun X, Rezac DJ. Effect of environmental noise and music on dexmedetomidine-induced sedation in dogs. PeerJ 2017;5:e3659.
- Lee AH, Detweiler KB, Harper TA, Knap KE, de Godoy MRC, Swanson KS. Physical Activity Patterns of Free Living Dogs Diagnosed with Osteoarthritis. J Anim Sci 2021.
- Mejia S, Duerr FM, Salman M. Comparison of activity levels derived from two accelerometers in dogs with osteoarthritis: Implications for clinical trials. Vet J 2019;252:105355.

96-97 – Serum glucose and insulin…
You repeat this information in the part on the insulin and glucose assays, I would recommend omitting this information in line 96-97.
AUTHORS’ response: We made the suggested change.

99 – this information is already stated before, I would delete one of the passages. You could, for instance, add the information on the tables earlier to line 86.

‘We recruited 22 dogs from four farms in the ….. [randomizer.org](http://randomizer.org)) (Table 1 and 2).
This way, you can avoid this information.

AUTHORS’ response: We made the suggested change.

“We recruited 22 dogs from four farms in the North Island of New Zealand and randomized the dogs into two balanced groups with respect to diet (n=11 each) using an online randomization tool (www.randomizer.org) (Tables 1 and 2). Dogs were recruited after receipt of each owner’s verbal consent to participate in the study. Dogs were only included in the study if they had been assessed by a physical exam by at least one of the authors and by the history provided by the owner and were deemed to be in good health” (L84-89).

101- Were any blood examinations, such as biochemistry or hematology, performed to assess for subclinical disease?
AUTHORS’ response: The short answer is no. The longer explanation is that dogs that are involved in high level of work such as these dogs cannot perform their work if they have underlying conditions and the owners quickly see that there is alteration in their performances. Also, it was not in our budget to prescreen with intensive bloodwork and additional imaging which would have been required in order to have confidence that the dogs do not have an underlying condition. We qualify our observations and wrote that the dogs were ‘deemed’ in good health and ideal body condition following physical exam and owner’s history.

106- You could delete this sentence, as you state this information more extensively below.
AUTHORS’ response: We made the suggested change.

116-122: I would state this in the discussion section, and not here. It would have been interesting to see whether the energy intake required to maintain bodyweight is different between both diets, but this would be a subject for further study.
AUTHORS’ response: We prefer to keep these sentences in the Diets paragraph within the Methods section (unless the reviewer would strongly object) for the reason that the reader immediately understands the rationale for why we chose to do what we did.

141-173 Suggestion: you could consider combining some paragraphs here, to avoid switching between insulin and glucose, activity levels and back to glucose and the HOMA-IR. You could combine the information of the assays, the IG glucose measurements and the HOMA-IR, and describe the triaxial accelerometry after this.
AUTHORS’ response: We combined the insulin and HOMA-IR paragraphs together and kept the FGM and triaxial accelerometry in one paragraph. We think that it keeps the flow better and the structure more coherent.

175- Please provide the full name of HOMA-IR also in the text before using the abbreviation.
AUTHORS’ response: We made the suggested change (L171)

192- How did you check for normal distribution? Could you provide any information about whether the assumptions of all tests where met?
Was any power analysis performed for this study?
AUTHORS’ response: We added the following sentence (L196-197) indicating how we assessed for normal distribution: “The data were examined for normal distribution by inspection of Q-Q plots, histogram, and by the Shapiro-Wilk test.”

Presence or absence of normal distribution was a criterion that determined whether ANOVA (normally distributed data) or nonparametric (nonnormally distributed data) tests had been used (indicated in L207-210).

We also added the following paragraph (L189-193): “A priori power analysis (G*Power software version 3.1.9.2) indicated that 9 dogs in each group will be sufficient to detect a difference between two dependent IG means of 100 mg/dL and 70 mg/dL in an individual dog with SD of 30 mg/dL, power of 0.8, alpha error probability of 0.05, and a correlation coefficient of 0.6 between measurements. To account for potential losses of dogs during the study period (i.e., dislodgement of the sensor), we recruited 11 dogs per group.”

The modelling of IG and activity using ASReml do not require the traditional assumptions to perform analysis of variance using the least-squares method (normality, homogenous variance, independent residual errors and additive effects), because the solutions of the model were obtained using restricted maximum likelihood procedures, which do not require the homogeneity of variance assumption; and the correlations among random residuals were accounted for in the modelling of observations for each dog using splines.

Results:

222-223 You also state this in your methods section, and could consider omitting it here.

AUTHORS’ response: We made the suggested change.

The results section itself may benefit from some editing to improve conciseness.

AUTHORS’ response: We trimmed a few sentences in different paragraphs of the results that were repetitive or redundant.

For instance, you could consider removing sentence 282, as you state this in the section heading.
AUTHORS’ response: We made the suggested change.

It is important to consider differences between the farms: did you consider including the factor ‘farm’ in your models?
AUTHORS’ response: We tested for a ‘farm’ effect when we analyzed the data and since ‘farm’ did not significantly affect the model it was omitted.

Discussion:
I think it is also beneficial for this report to consider the effects of the other macronutrients of the diets, which also differed widely. The diets also differed widely in dietary fat and protein content, two factors which have also been found to affect glucose tolerance and insulin resistance. It is therefore a possibility that the effects that you found are not due to a lack of carbohydrate content, but maybe an increase in dietary fat content.
AUTHORS’ response: The reviewer is correct. Throughout the revised manuscript we referred to Diet 2 as “ultra-low CHO high fat” and Diet 1 as “high CHO low fat” as they are linked together and decreasing either CHO or fat in the diet leads to reciprocal increase in the other component. While the diets also differed in %ME protein we do not think that this difference [23% (Diet 1) vs 37% (Diet 2)] had the same effect as the marked differences in CHO and fat. In the discussion we also ascribe the dietary effect that we found in our study to the process of fat adaptation.

Also, differences in housing/environment of the dogs on the different farms may have also affected your results. As an example: maybe the dogs of one of the farms were housed in colder conditions, and had a higher energy requirement (and where therefore more likely to be hypoglycemic?)
AUTHORS’ response: The four farms in the study were from the Waikato region in New Zealand. Housing conditions of farm dogs in New Zealand are quite standard (outdoors kennel). Therefore, we think that it is very unlikely that the dogs in the different farms were subjected to very different terrains or ambient temperatures. Also, as we knew that we could not completely control for an identical workload between farms (although we think the workloads were quite similar given we aimed the 96-hour periods to occur at peak seasonal activity) we made sure to balance the diet within the farms. Hence, despite the inherent minimal differences between farms in ambient temperature, terrain, and workload we think that the major effects on IG and activity are foremost due to the diet.

Tables and figures:
- Table 1: I think this table may be improved by swapping the ‘Farm’ and ‘variable’ columns. Additionally, I would suggest splitting these tables, as the last two rows do not fit the table headings.

AUTHORS’ response: We made the suggested change.

- In table S1, the nutritional analysis of diet 2 is missing.

AUTHORS’ response: We added the nutritional analysis of diet 2 that was accidently deleted.

Also, is there any information about the specific nutrient values of both diets (for instance, omega-3 fatty acid concentrations, vitamins and minerals)? I think it is important to provide this information, considering omega-3 fatty acids and omega 6:3 ratios have been found to alter insulin sensitivity, and therefore may affect your results.
AUTHORS’ response: A full nutrient profile for commercial diets is $2,500 and beyond the budget of this trial, thus unfortunately, we don’t have the full nutritional analysis of each diet. That is proprietary information, and we did not have access to that, beyond what information was included in the tables for Tux (Diet 1). A little more was available for the K9 Natural product (Diet 2), but not for both diets.

Within the scope of AAFCO requirements, we are not aware that insulin sensitivity will be affected by micronutrient concentrations in a manner that exceeds the effect from the massive difference in CHO content.

The effect of n-3 PUFA has been shown in diabetic humans. The effects of n-3 PUFA in healthy dogs are less clear. One study showed that enrichment of the diet with n-3 PUFA at 0.2% (as fed basis) had no effect on insulin sensitivity (Irvine et al 2002), and another showed no relationship between serum n-3 PUFA concentrations in dogs and serum insulin (when the dogs were eating various unreported diets), whilst obesity did have a significant effect.

The ultra-low CHO diet (Diet 1) contains a fish oil (Hoki oil), and the n-3 PUFA content is 0.28% (as fed basis), and thus is almost exactly the amount tested by Irvine et al (2002). The dry diet contains a small amount of the n-3 alpha linolenic acid, but no significant amount of long chain n-3 PUFA, and thus would be similar to the control diet in the study by Irvine et al.

We think that there is no reason to suspect that the different PUFA contents of the diets have a significant effect on insulin sensitivity. Compared with the effect of the difference in macronutrients (i.e. CHO, fat, and protein), any effect from micronutrient differences is argued to be small.

Streeter RM, Struble AM, Mann S, Nydam DV, Bauer JE, Castelhano MG, Todhunter RJ, Cummings BP, Wakshlag JJ. The associations between serum adiponectin, leptin, C-reactive protein, insulin, and serum long-chain omega-3 fatty acids in Labrador Retrievers. Vet Med (Auckl). 2015 Apr 8;6:103-110.

Irvine AJ, Butterwick R, Watson T, Millward DJ, Morgan LM. Dietary supplementation with (n-3) polyunsaturated fatty acids does not affect insulin sensitivity in healthy Labrador retriever dogs. J Nutr. 2002 Jun;132(6 Suppl 2):1709S-10S

Thank you in advance for your response.

AUTHORS’ response: Thank you for providing the feedback required to improve and better our manuscript.
