# Supplementary material for: Less is more? Ultra-low carbohydrate diet and working dogs’ performance
Source: PLoS One. 2021 Dec 23;16(12):e0261506. doi: 10.1371/journal.pone.0261506 (PMC8699952; doi:10.1371/journal.pone.0261506)
Supplement: Attachment — Submitted filename: Response to Reviewers.docx [file pone.0261506.s008.docx]

PONE-D-21-02404R: Less is more? Ultra-low carbohydrate diet and working dogs' performance

The authors thank the Editor and both Reviewers for the time they invested in reviewing the manuscript and providing their valuable comments and suggestions for improving the manuscript and making it more impactful. Below we provide a point-by-point responses to each of the comments made by the Editor and Reviewer 2.

**REVIEWER 1**

Authors’ response: Thank you!

**REVIEWER 2**

Reviewer #2: Overall, a very interesting manuscript. Additional work investigating the effect of nutrition on active, working dogs is much needed. My biggest concern is about statistical analysis of the data, which is outlined in the methods section below.

Authors' response: After we went through the rebuttal process, we think that the main problem that Reviewer 2 had with our statistical analysis stemmed from a problem with terminology on our end for which we apologize. Specifically, we think that the use of ‘activity’ and ‘activity level’ interchangeably confused Reviewer 2 with regards to what was used as a response variable and what was used as a fixed effect in our analyses. As we explain below, we therefore used ‘delta-g’ as a response variable (derived from the accelerometer recordings), and we stratified ‘delta-g’ to three levels (L/M/H) of activity to assess the effect of ‘activity level’, ‘diet’, and ‘time’ (across the 96-hour of data recording and split between ‘working period’ and ‘resting period’) and interactions between diet x time on IG.

Abstract:

L29 – Sentence reads awkwardly. The dogs were randomized to a diet then fed for a month, but the sentence almost reads as if the randomization took a month.

Authors' response: We changed the sentence as follows (L29): "At each farm, dogs were randomized to one of two diets and had a month of dietary acclimation to their allocated diet. "

L31 – Remove additional parenthesis around "Diet 1"

Authors' response: The parenthesis was removed.

Introduction:

General comment – Introduction would benefit from an additional paragraph on why a low CHO diet is selected. The second paragraph of the introduction describes a need for CHO during intense exercise, but then why are low CHO diets used at all? In the discussion, the focus shifts to how low CHO, high fat diets may be advantageous, but this should be introduced in introduction.

Authors' response: There are two primary feeding practices for working dogs in New Zealand. The reasons why these are the two common practices are multifactorial and involves farmers' personal beliefs and financial concerns (commercially available diets are generally more expensive than home-kill meat-based homemade diets) (L53-56).

We added the following in L56: “Dogs do not require dietary CHO, and exercising sled dogs may have improved performance when fed a high-fat CHO-free diet [2]. However, the concept of “cross-over”, where muscle utilization of CHO for ATP production increases with increased exercise intensity, has been demonstrated in several different mammalian species, including dogs [3]. Since the intensity of activity engaged by working farm dogs in New Zealand is likely to be greater than the sustained moderate-intensity endurance activity of sled dogs, New Zealand’s working farm dogs may be unable to maintain normal body glucose levels when fed a low CHO diet.”

Reviewer 1 was already concerned about the overall length of the manuscript and we are reluctant to add additional information beyond this.

L55 – Remove extra space in "(%ME )"

Authors' response: we removed the extra space.

L56 – Suggest expanding on why this is an important area of study (it is more than it being unknown)

Authors' response: We added the following in L56: “Dogs do not require dietary CHO, and exercising sled dogs may have improved performance when fed a high-fat CHO-free diet [2]. However, the concept of “cross-over”, where muscle utilization of CHO for ATP production increases with increased exercise intensity, has been demonstrated in several different mammalian species, including dogs [3]. Since the intensity of activity engaged by working farm dogs in New Zealand is likely to be greater than the sustained moderate-intensity endurance activity of sled dogs, New Zealand’s working farm dogs may be unable to maintain normal body glucose levels when fed a low CHO diet.”

L72-76 – Suggest switching the hypothesis and study aims sentences to first describe study aims then hypothesis/expected outcome

Authors' response: We would prefer not to do so unless the Reviewer/Editor is adamant that we change the order. Our rationale is that the aims are derived from the hypothesis and not vice versa.

Materials and Methods:

L85 – Please give additional information regarding how groups were randomized. Was the randomization balanced across farms, sex, and breed? It does not appear so based on Table 2. It may be simply due to the dogs available at each farm, but please state that here if that is the case.

Authors' response: The randomization was based on balancing the diets across farms and dog availability and not on sex, age, breed, or bodyweight because it was challenging to find farms and farmers that were willing to participate. Hence, we were unable to be too selective.

The sentence was changed as follows (L91): "We recruited 22 dogs from four farms on the North Island of New Zealand and randomized the dogs into two balanced groups with respect to diet (n=11 each) using an online randomization tool (www.randomizer.org); there was no attempt to balance the dogs per age, sex, breed, or bodyweight during randomization and recruitment (Tables 1 and 2)."

L86 – Italicize "n"

Authors' response: we italicized it.

L88 - Within what time period were the dogs assessed? Within weeks? A year?

Authors' response: One of the authors (WC) has been providing regular veterinary care to the farmers in this region. It is important to mention that WC gauged the interest of the farmers to participate in the study and enroll their dogs with respect to the high level of work those dogs were expected to do in the month to come. WC assessed the dogs on the farms of the farmers that indicated they were keen to participate and determined if the dogs were deemed appropriate to be enrolled in the study. Once WC decided to enroll the dogs, they were enrolled within days (i.e., the time it took us to ship the diets to the farmers in those farms). Then the acclimation period started and a month after that, the 96-hour of data recording commenced, which took place at the peak seasonal workload.

L90 – Remove extra space between "which" and "took"

Authors' response: we removed the extra space.

L94 – Suggest removing first person here and throughout - "Bodyweight was recorded at recruitment, then at the beginning and end of each of the 96 h periods."

Authors' response: We prefer to use an 'active voice' rather than a 'passive voice' as much as possible. This is a stylistic preference, and the Journal does not have a specific guideline regarding it. Unless the Reviewer is adamant about that, we prefer to leave it like that. The Editor may also have a preference to which we would defer. We think that there has been some shift in the style of scientific writing for the purposes of clarity and because English is a universal communication language, we want our writing to be easy to understand to a wide range of first and second language speakers.

L97 – When was the accelerometer fitted? At recruitment? Was there a baseline activity collection period?

Authors' response: We fitted BOTH the accelerometer and Freestyle Libre at the same time; it was at the end of the one-month dietary acclimation period and at the beginning of the 96-hour data recording period. The 96-hour data recording period was at the PEAK OF SEASON ACTIVITY (see our previous comment for L88). So, there was no baseline recording period. We fitted the dogs with the devices, collected the blood (baseline samples), and off they went to work.

L126 – Serum tubes allowed to clot before what? Assuming this means allowed to clot before being put on ice since the samples were not processed on site, but please clarify.

Authors’ response: We added the word ‘Then’ (L136) and we hope that it is now clear: “Whole blood in the serum tubes was allowed to clot for 10 minutes whereas the whole blood in the sodium fluoride tubes was inverted several times to allow for the anticoagulant to mix with the blood. Then, the samples were stored on ice as long as the veterinarian was in the field and shipped on ice overnight to the principal investigator."

L136 – Remove comma after "whereas"

Authors' response: We removed the comma.

L149-150 – Why no baseline measurement during month of acclimation?

Authors' response: There are two reasons for that. We simply did not have the resources and were working on a tight budget (we paid per the time the accelerometer recorded; also, each FSL sensor is quite expensive and has a maximum lifetime of two weeks, albeit it works for a much shorter time in most dogs). Also, it is one thing to ask the busy farmers to flash the readers over the sensors at least every 6 hours for 96 hours and a different thing to do so for a more extended period. Compliance, recruitment, and logistics were significant hurdles in this study. The second reason is that there was a big difference in the farm workload between the beginning of the acclimation period and the beginning of the 96-hour data recording period. We agree that it would have been interesting to have this data and make comparisons. But we do not think that not having it changed our ability to answer our study question.

L159-160 – Please include any relevant citations for established blood glucose levels

Authors' response: We inserted a citation from a veterinary clinical biochemistry book (L168) that has a population-based reference interval for canine blood glucose.

L168 – Please provide inter- and intra-assay variation and sensitivity for this assay

Authors' response: We added the requested information (L178): “The assay’s lower limit of detection and range are 0.5 µU/L and 1-300 mU/L, respectively. The within run coefficient of variation (CV) at insulin concentrations of 27 mU/L and 65 mU/L are 1.05% and 3.9%, respectively. The between run CV at insulin concentrations of 27 mU/L and 65 mU/L are 4.07% and 6.83%, respectively.”

L171 – Suggest brief explanation of what HOMA-IR is. What is normal? What is expected in this population of dogs?

Authors' response: We added the following (L182): “The homeostatic model assessment of insulin resistance (HOMA-IR) is an epidemiological tool used in people to assess insulin resistance. High HOMA-IR levels indicate an increased resistance to insulin, and lower levels indicate an increased sensitivity to insulin’s action. The HOMA-IR was calculated as previously described [17, 18] according to the following formula: plasma insulin x serum glucose / 22.5.”

L174-177 – I do not see the purpose of this arbitrary distinction into activity levels, particularly with so few animals who were all highly active working dogs. This becomes a problem later on in the statistical analysis section when activity (a response variable) is used as a fixed effect for IG analysis.

Authors' response: stratification to three levels of activity allowed us to see the diet x activity level interaction on IG levels. As the Reviewer can see, there is a different metabolic ‘behavior’ at low/moderate levels of activity between the high and low CHO diets. This opens an intriguing question of why this is happening and what are the underlying physiologic mechanisms involved. Furthermore, as the Reviewer can see, the level of IG for the low/moderate activity levels in the high CHO diet is not different than that of the high level of activity in the low CHO diet. Reenforcing the argument above, it possibly suggests different utilization of energy resources for metabolism (i.e., fat vs. CHO) between the diets (and possibly between different levels of activity). Lastly, stratification of activity to three levels also allowed us to determine in what level of activity the dogs are more likely to have low glucose readings (an interesting question on its own).

L176 – Is the =< supposed to be ≤ or

Authors' response: We changed the signs to be consistent with the Reviewers.

L198-199 – Were data transformations attempted?

Authors' response: Variables that did not follow normal distributions were analyzed with non-parametric tests to avoid the transformation and provide clear biological explanation on a nominal scale. We think that it is a valid statistical approach.

L201-203 – several questions:

- Why is time not included as a fixed effect?

Authors' response: Thank you for this important comment. Following your comment, we included ‘period’ as a fixed effect after converting the 96 hours into an 8-level categorical variable, ran the model again, and the only thing that changed is that there was no difference between the low and moderate levels of activity within diet 1 (high CHO low fat). Fig 3 was revised to reflect this change. This is now stated in L213-220.

- Above, you mention activity and inactivity periods, but these are also not reflected in this analysis

Authors' response: To prevent confusion, we changed ‘activity period’ and ‘inactivity period’ to ‘working period’ and ‘resting period’, respectively. The description for the statistical analysis of the frequencies of low vs. normal/high IG readings during the ‘working’ and ‘resting’ periods was explained in L241.

- What about farm, sex, age, breed, etc.? If these were evaluated as fixed effects then dropped, that should still be documented here.

Authors' response: We explored each one as a single effect and dropped from the final model because they were not significant.

- Activity is a response variable, not a fixed effect. If you wanted to include "activity level" as a fixed effect, you would need baseline activity data by which to block the dogs by activity level prior to diet allocation & acclimation

Authors' response: We did not analyze activity *per se*, but we used delta-g as a measure of activity. We think that it created a confusion (for which we apologize) and we corrected the manuscript to indicate that the dependent variables were IG and delta-g (not activity as you correctly indicated). The study was designed to assess the effect of diet on the level of IG and delta-g as a measure of activity. The classification by L/M/H activity levels enabled us to find biologic/metabolic changes in IG that are related to the interaction between diet x activity level.

In order to establish a ‘baseline’, we would have had to fit the dogs with accelerometers and FSL sensors at the beginning of the acclimation period, which as we explained above in response to one of your previous comments (L149-150) was not feasible financially and logistically. We think that the procedure to classify dogs as having low, moderate, and high activity levels was the best way we could classify the dogs. We think that our approach is valid for answering the research question.

L207-208 – Again, were any transformations attempted prior to analysis as a nonparametric variable?

Authors' response: Variables that did not follow normal distributions were analyzed with nonparametric tests to avoid the transformation and provide clear biological explanation on a nominal scale. We think that it is a valid statistical approach.

L208 – Remove comma after "whereas"

Authors' response: We removed the comma.

L209 – Why GLM here but MIXED above? What was included in the GLM model?

Authors' response: Thank you again for your comment. To be consistent, we changed the analysis from GLM to the MIXED procedure and described the MIXED model more precisely (L226).

L213 – Why not include time & diet x time interaction here?

Authors' response: Thank you for the suggestion. We modeled the curves of activity and IG through the time for each dog. This description of the model was modified in the text. To further explain, the modeling of the curves for level of glucose and the delta-g were done at the level of the dog. Later, we wanted to obtain the average curve of level of glucose and delta-g for each of the diets, which was achieved by averaging individual dog’s splines coefficients to achieve the mean and standard error of the coefficients for each diet. The interaction between diet and time can be evaluated but it is difficult as in this case time is a continuous variable. From Fig 2 it is seen that the level of glucose is different across time for each of the diets. If diet and time interaction would have been tested, they would have been significant. However, it was hard to achieve it because the use of splines. If the modeling of the dependent variables IG and delta-g would have been done with a polynomial the interaction could have been achieved but we chose not to use a polynomial because a polynomial will not describe adequately the variation for these variables.

L214 – Is "activity level" here referring to the "activity levels" described above (L174-177)? Why examine activity level as a dependent variable here but a fixed effect in the linear models above?

Authors' response: We apologize for the confusion. We changed the text to IG and delta-g which refers to the dependent variables that were modeled.

Results:

L221 – How was BW statistically analyzed? This is not described in methods. The description of BW effects is a bit clunky. Were diet and time fixed effects significant? The interaction? You jump straight to describing the individual diet x time interactions without presenting results of main effects.

Authors' response: We added the description of analysis of BW in the methods L232 and the results are presented in the result section L259.

L225 – Suggest authors use consistency with respect to P-value reporting – 2 decimals used here versus three in next line. Recommend confirming if journal has standard for reporting P-values.

Authors' response: We changed all P values to include 3 decimal places.

L226 – With the standard errors reported here, these weights should not have a statistical difference. Something is not correct with this analysis.

Authors' response: Thanks for this observation which led us to review and revise this part of the statistical analysis in the statistical methods. The LSM were similar but the standard error changed which make the results significantly different.

L229 – What about time effect? Diet x time interaction?

Authors' response: Thanks for this important point! The modeling of the curves for level of glucose and the delta-g were done at the level of the dog. Later, we wanted to obtain the average curve of level of glucose and delta-g for each of the diets, which was achieved by averaging individual dog’s splines coefficients to achieve the mean and standard error of the coefficients for each diet. The interaction between diet and time can be evaluated but it is difficult as in this case time is a continuous variable. From Fig 2 it is seen that the level of glucose is different across time for each of the diets. If diet and time interaction would have been tested, they would have been significant. However, it was hard to achieve it because the use of splines. If the modeling of the dependent variables IG and delta-g would have been done with a polynomial the interaction could have been achieved but we chose not to use a polynomial because a polynomial will not describe adequately the variation for these variables.

L242 – Your hypothesis is that low CHO diets lead to low IG which in turn reduces activity. Now you are trying to attribute activity to IG levels! Again, activity is a response variable, not a fixed effect. Dogs' activity is likely related to IG, but that is not what this study was designed to assess. This section should be removed. “activity is a response variable, not a fixed effect”

Authors' response: We did not analyze activity *per se*, but we used delta-g as a measure of activity. We think that it created a confusion and we corrected the manuscript to indicate that the dependent variables were IG and delta-g (not activity as you correctly indicated). The study was designed to assess the effect of diet on the level of IG and delta-g as a measure of activity. The classification by L/M/H levels of activity enabled us to find biologic/metabolic changes in IG that are related to the interaction between diet x activity level.

L256 – Period of the day is not included in your statistical analysis (L201-203) as a fixed effect.

Authors' response: Good observation. We revised the description of the statistical analysis of the frequencies of low vs. normal/high IG readings during the ‘working’ and ‘resting’ periods, which is now explained in L241. We looked at frequencies of low vs. norm/high IG and used the Chi Square test to determine if there were differences.

L256 – Is the incidence of low IG more relevant than time spent at low IG? How much total time was spent at low IG? The potential diet x time interaction is important.

Authors’ response: The incidence of low glucose is important as well as the overall time of low IG. The incidence is important because until recently when veterinarians started to use the FSL (FreeStyle Libre) we did not think that healthy animals have low IG readings. We are saying it carefully because we do not have reference intervals for IG (as we discussed it in length in the discussion). The point is that now we know (from this study and growing clinical experience) that healthy animals could have low IG readings without clinical signs of neuroglycopenia. How many times a dog has low IG is important because its sympathoadrenal responses should prevent it from happening too often. If the incidence starts to increase above a certain level (which is yet to be defined) then there might be a physiologic or clinical problem. However, the ‘normal’ incidence and frequency of low IG in dogs is unknown and this study would be the first to show it. At the same note, how long a dog has low levels of IG is also important. If there are prolonged periods of low IG, the probability of development of clinical signs related to neuroglycopenia increases as well as the chance that an underlying physiologic or clinical problem exist.

In L312 we described that median (range) of low IG events is 6.5 (IQR 17.5; range 1-42). A median of 6.5 equals to 6.5 x 15 min = 97.5 min per the 96-hour of data recording. You can see that one dog spent 10.5 hours (not continuously) per the 96 hours of data recording with low IG level. This dog as much as we know has been completely normal and did not have any clinical problems or changes in its quality of work (in NZ working dogs are “decommissioned” if they have a decrease in work quality).

We analyzed the interaction of time x diet when IG is low by doing a Chi-Square test and found it to be significant (p < 0.001). The result is now indicated in the text in L308.

L266 – Remove extra space between "Diet 2" and "group"

Authors’ response: Extra space removed.

L269-270 – Would it be expected for the majority of low IG readings to occur in the activity period? Would be nice to mention whether or not this was expected.

Authors’ response: Yes, we expected that most low IG would be during the working period. We prefer to discuss our expectations and interpretation of the results in the discussion rather than in the result section.

L275 – Remove comma after "whereas"

Authors’ response: Extra space removed.

L281 – Again, activity is not a fixed effect, and your hypothesis has the reverse – low IG reducing activity. This section should be removed.

Authors' response: We did not analyze activity *per se*, but we used delta-g as a measure of activity. We think that it created a confusion and we corrected the manuscript to indicate that the dependent variables were IG and delta-g (not activity as you correctly indicated). The study was designed to assess the effect of diet on the level of IG and delta-g as a measure of activity. The classification by L/M/H activity levels enabled us to find biologic/metabolic changes in IG that are related to the interaction between diet x activity level.

L299 – Consider re-wording as there is no such thing as "inappropriate" serum insulin.

Authors’ response: The sentence was changed to “None of the dogs had evidence of hyperinsulinemic hypoglycemia…” (L338)

Discussion:

L304 – These results were presented above, the reference to the figure is not needed here.

Authors’ response: We started the discussion with the statement of the most important finding in the study and developed the paragraph over this statement. It was specifically requested by Reviewer 1 during the previous revision and we made extensive changes in the structure of the discussion to accommodate Reviewer 1’s request. We think that it is legitimate to refer to the figures in the discussion. If the editor would require removing the reference to the figures in the discussion, we will remove it.

L307 – Suggest removing "most"

Authors’ response: We removed ‘most’

L314 – Reword "people" as "humans" throughout

Authors’ response: The word ‘humans’ substituted the word ‘people’ throughout the manuscript.

L321-322 – Where is it suggested that low fat/high CHO diet offers advantages? Your hypothesis is that lower CHO/high fat diet will reduce activity.

Authors’ response: The word ‘above’ in L360 refers to the beginning of this paragraph where we discuss the advantages of adaptation to a high fat low CHO diet.

L323-326 – These two sentences are very repetitive. Suggest condensing into one sentence.

Authors’ response: We condensed the two sentences to one (L362) “For example, exhaustive exercise in sled dogs that consumed a diet with 0% of metabolizable energy from a CHO source and high %ME from fat had higher albumin, total calcium, and magnesium than control dogs on diets with higher %ME from a CHO source [27].”

L334 – Reword to "necessarily"

Authors’ response: The word ‘necessary’ was removed.

L339-340 – Here you could discuss how activity could have an impact on IG and recommend additional research since your study was not designed to examine this effect.

Authors’ response: At the end of the paragraph we added the following L385: “However, further studies are required to provide a mechanistic insight to determine whether either or both of the above explanations, or alternative explanations underly the temporal relationship between delta-g and IG as our study was not designed to investigate it.”

L340 – Remove reference to figures from discussion

Authors’ response: We think that it is legitimate to refer to the figures in the discussion. We will refer to the Editor’s decision about this point.

L347 – Suggest further discussion at the end of this paragraph as to the potential significance of this observed relationship.

Authors’ response: We do not think that in isolation, the temporal relationship that we described in this paragraph, has a significant implication as both 1) increased glucose production by the liver and reduced glucose utilization by insulin-dependent tissues at high activity levels, and 2) increased muscle and liver glucose uptake immediately after low activity levels have been described in dogs. We think that the pronounced fluctuation in IG and a lower AUC glucose in Diet 2 vs. Diet 1, combined with the fact that dogs in Diet 2 had higher AUC delta-g is really the interesting finding here that has a biological significance and we discussed it in the first paragraph.

L349-350 – Awkward wording, suggest re-working. Since the hypothesis is that low IG leads to reduced activity, suggest focusing on that. May discuss potential for higher activity to reduce IG as avenue for future research, but not really relevant here.

Authors’ response: The sentence was rephrased as follow (L390): “During the 96 h of data recording there were neither low peaks of IG that followed high peaks of delta-g, nor low peaks of delta-g followed low peaks of IG.” What we are saying is that the temporal relationship seen in Fig 2 was that low IG always followed low delta-g and that we think that it was because of the large flux of glucose into the liver and muscle as dogs started to rest after high activity level. We discussed it in the previous paragraph and this paragraph basically indicate that they did it in high efficiency because they were very sensitive to insulin.

L351 – Expand on the potential limitation of the 96 h collection period. What time period would have been better?

Authors’ response: This is a hard question to answer and we do not have an answer. It maybe never if the explanation that we gave is correct (i.e., low IG followed low delta-g because large flux of glucose into the liver and muscle occurred as dogs started to rest immediately after periods of high activity level) or a slightly longer or much longer than 96 h if low peaks of IG would follow high peaks of delta-g, or low peaks of delta-g would followed low peaks of IG.

L358 – Suggest "of this alternative"

Authors’ response: We made the requested suggestion (L395)

L360-361 – Suggest removing "The incidence of low IG episodes was 3.12% per 96 h and involved 10/22 (45.5%) of the dogs" from the discussion since it has already been reported in results section.

Authors’ response: We deleted the sentence.

L363 – Similar to comment in L299 above, please remove "inappropriate" and re-word.

Authors’ response: L401 now reads: “The dogs in this study had their serum insulin and glucose concentrations measured at the beginning and the end of the 96 h study periods, and none of the dogs had results that were suggestive of hyperinsulinemic hypoglycemia.”

L364 – Suggest removing "we conclude that"

Authors’ response: The sentence now reads as follows (L404): “Therefore, healthy dogs could have a low incidence of low IG and the frequency of low IG may be greater in dogs fed ultra-low CHO high fat diet (Diet 2).”

L365 – Is the fact that healthy, active dogs can have low IG really a surprising conclusion/finding of this study? Suggest writing different sentence to link discussion between serum insulin/glucose and IG

Authors’ response: Yes, it was a surprise. Having low levels of glucose of this magnitude has been thought of as an abnormal or pathological condition. Seeing that normal healthy dogs could have it on occasion changes the perspective about finding low IG in dogs. As we eluded, low IG ≠ low blood glucose (BG) especially when you do not have reference intervals. Apparently, there could be many things that can induce low IG and this study identified diet and activity as two factors that were related to it.

L373 – What effect might feeding time/frequency have on IG?

Authors’ response: Interesting question! If dogs are not adapted to alternative sources of energy (i.e., fat) then we speculate that low glucose levels would be more likely to happen as the time elapsing between the incidence of low glucose and feeding increases. This could aggravate if the dog is under any chronic stress (physical, pathological, mental). On the other hand, low levels of glucose could also happen postprandially if the incretin-mediated insulinemic effect is not met with a matching opposing effect mediated by glucagon ± cortisol.

L377 – How does the evening feeding time play into this? Suggest further discussion

Authors’ response: We do not know the answer to this question. We explain just above how low levels of glucose might happen postprandially. But there was a strong diet related effect, so it is not simply a timing issue. We think that we discussed it adequately in this paragraph.

L409 – Any suggestions for future investigation regarding IG monitoring? Potential future work could be worked in throughout discussion.

Authors’ response: To know, one would need to perform invasive studies in dogs that are way beyond the scope of this study. These would include catheterization of the portal and hepatic veins to measure pattern of pancreatic hormone secretion and hepatic glucose output in dogs that will be on high CHO low Fat vs. low CHO high fat combined with EEG monitoring of sleep patterns, IG monitoring, BG monitoring, and VO2 monitoring in combination with periods of strenuous activity (on a treadmill or alike) and rest. This is just not something you can achieve with a simple field study such as this.

L418 – Suggest addition of how this limitation could be overcome, such as formulating the diets to be similar in every respect except for source of energy (CHO vs. fat)

Authors’ response: We added the following in L457: “A future study could reduce nutritional confounders by formulating the diets using varying proportions of the same ingredients, such that only the macronutrient proportions differ.”

Conclusion:

L435 – Spell out abbreviation when it begins a sentence.

Authors’ response: (L476) we spelled out interstitial glucose.

Tables and Figures:

Fig. 1 – in caption, should read "an ultra-low CHO…" not "a"

Authors’ response: (L85) we revised to "an ultra-low CHO…"

Table 2 – Diet randomization is clearly not balanced across farms, particularly farm 3. If there is a reason for this, please include in methods section.

Authors’ response: We did our best to balance the diets across the four farms. In farm 3 there was a problem and it was not as balanced as in the other farms. Obviously now we cannot do anything about it but looking at the variables in Tables 1 and 2 one can tell that the randomization process was successful because there were no significant differences in the demographics across farms and diets.

Table 3 – Recommend removal since activity should not be used as a fixed effect.

Authors’ response: We assume the Reviewer meant Fig 3 as the manuscript does not have Table 3. We explained above that we did not analyze activity *per se*, but we used delta-g as a measure of activity. We think that it created a confusion and we corrected the manuscript to indicate that the dependent variables were IG and delta-g (not activity as you correctly indicated). The study was designed to assess the effect of diet on the level of IG and delta-g as a measure of activity. The classification by L/M/H levels of activity enabled us to find biologic/metabolic changes in IG that are related to the interaction between diet x activity level. Fig 3 is important because it shows the interaction between activity level and diet with respect to IG. As we explained before, the stratification to three activity levels allowed us to see the diet x activity level interaction on IG levels. There was a different metabolic ‘behavior’ at low/moderate levels of activity between the high and low CHO diets. This opens an intriguing question of why this is happening and what are the underlying physiologic mechanisms involved. Furthermore, as the Reviewer can see, the level of IG for the low/moderate activity levels in the high CHO diet is not different than that of the high level of activity in the low CHO diet. Reenforcing the argument above, it possibly suggests different utilization of energy resources for metabolism (i.e., fat vs. CHO) between the diets (and possibly between different levels of activity). Lastly, stratification of activity to three levels also allowed us to determine at what activity level the dogs are more likely to have low IG readings (an interesting question on its own). We request to keep the figure from these reasons.
